# Bleeding Complications in JAK2‐Variant Essential Thrombocythemia: A Revisit in 2025

**DOI:** 10.1002/jha2.70088

**Published:** 2025-07-04

**Authors:** Gerard Gurumurthy, Samantha Gurumurthy, Tim C. P. Somervaille, Anna Falanga, Jecko Thachil

**Affiliations:** ^1^ The University of Manchester Manchester UK; ^2^ Department of Infectious Disease Imperial College London London UK; ^3^ Cancer Research UK Manchester Institute The University of Manchester Manchester UK; ^4^ The Christie NHS Foundation Trust Manchester UK; ^5^ Department of Transfusion Medicine and Hematology Hospital Papa Giovanni XXIII Bergamo Italy; ^6^ MAHSC Professor The University of Manchester Manchester UK

## Abstract

Essential thrombocythemia (ET) is a myeloproliferative neoplasm characterised by sustained thrombocytosis. Paradoxically, bleeding complications remain an under‐recognised clinical challenge. Compared with CALR‐mutated patients, those harbouring the JAK2‐V617F variant appear more prone to haemorrhage. This may be secondary to acquired von Willebrand syndrome (AvWS) and intrinsic platelet dysfunction. AvWS in ET arises from extreme platelet counts driving the adsorption and proteolysis of high‐molecular‐weight von Willebrand factor (VWF) multimers, producing a qualitative VWF defect akin to type 2A von Willebrand disease. However, the platelet count threshold for AvWS is variable, and patients with platelet counts below 1000 × 10^9^/L may still exhibit clinically significant VWF anomalies. Diagnosis relies on tests such as VWF activity and antigen levels. Perioperative management of patients with AvWS focuses on correcting the VWF defect and/or lowering platelet counts. Ultimately, clinicians must balance between preventing thrombosis and bleeding by tailoring management to each patient.

**Clinical Trail Registration**The authors have confirmed clinical trial registration is not needed for this submission.

## 1 Introduction

Myeloproliferative neoplasms (MPNs) are clonal hematopoietic stem cell disorders characterised by the overproduction of mature blood cells. Essential thrombocythemia (ET) is an MPN marked by sustained thrombocytosis and increased numbers of enlarged, mature megakaryocytes with hyperlobated nuclei in the bone marrow [1]. It is relatively rare, with an incidence of roughly 1 to 2 new cases per 100,000 people per year and an estimated prevalence of around 40–60 per 100,000 [2]. Genetically, 50–60% of patients harbour a JAK2 variant (most commonly JAK2 V617F), 25−30% a CALR variant, and a smaller fraction have an MPL variant. 10–20% have none of these “driver” variants (so‐called “triple‐negative” ET) [3, 4, 5, 6]. Driver mutations influence disease phenotype and complications. In particular, JAK2‐variant ET tends to present with higher haemoglobin and leucocyte counts, reflecting the greater erythroid and granulocytic bias induced by the JAK2 V617F variant. Relatedly, over 15 years, up to 30% of patients initially diagnosed with JAK2‐variant ET, may subsequently meet diagnostic criteria for polycythaemia vera. JAK2‐variant ET also has a well‐documented higher risk of thrombosis [7]

### 1.1 Bleeding Risk in Essential Thrombocythemia

Paradoxically, despite the hallmark thrombocytosis, patients with ET also experience bleeding complications. Bleeding in ET can range from minor mucocutaneous bleeding (such as frequent nosebleeds, easy bruising or gum bleeding) to major haemorrhages like gastrointestinal (GI) bleeds or intracranial haemorrhage [8, 9, 10, 11]. The incidence of major bleeding events (defined as fatal bleeding, critical site bleeding, a drop in haemoglobin > 20 g/L or bleeding requiring transfusion of ≥ 2 units [12]), in ET is not well‐defined (Table 1). An observational study found that major bleeding events occurred at a rate of 0.79% per year [13], while other sources have estimated the overall lifetime risk to lie between 5% and 30% [14]. In a large series of 565 patients with ET, the cumulative risk of a haemorrhagic complication (defined as life‐threatening and/or requiring one or more units of red cells/day) was 14% at 10 years [15]. Notably, the gastrointestinal tract is the most common site of major bleeding in ET, accounting for about half of cases [8]. These bleeding complications contribute to the increased morbidity risk of patients with ET. One study suggested that bleeding has a higher risk of mortality in ET than thrombosis [10]. This dual risk has long complicated the management of ET. This challenge is further compounded in those harbouring the JAK2 V617F variant who are noted to be at an increased risk of bleeding and thrombosis compared with patients with other variants [16, 17].

**TABLE 1:** Studies reporting on bleeding incidence/risk in essential thrombocytopenia.

Study	Study Design	Definition of Bleeding	Bleeding Incidence	Comments
Finazzi et al. (2012)	Comparing bleeding risk and incidence in ET and prefibrotic myelofibrosis	Major bleeding: defined as a symptomatic haemorrhage in a critical organ or an overt haemorrhage requiring transfusion or associated with a haemoglobin decrease >20 g/L without transfusion	Incidence of major bleeding: 0.79% patients/ year	Sample size: 891 ET patients Median follow‐up: 6.2 years
Nicol et al. (2021)	A systematic review of bleeding in ET and polycythaemia vera	Major bleeding: ISTH definition (fatal, critical site, haemoglobin drop ≥2 g/dL, or transfusion of ≥2 units)	Median incidence of all bleeding: 4.6% per year Median incidence of major bleeding: 0.79% per year	Sample size: 10370 ET patients Median follow‐up: 5 years
Palandri et al. (2012)	Retrospective analysis to identify bleeding characteristics	Severe bleeding: defined as a life‐threatening bleeding event or an overt haemorrhage requiring one or more units of red cells per day	Cumulative bleeding incidence: 11% Cumulative severe bleeds: 4% Incidence rate: 13.9 bleeds per 1000 person‐years (all bleeds), 5 per 1000 person‐years (severe bleeds)	Sample size: 565 ET patients Median follow‐up: 7.8 years 77% (20) of major bleeding patients were on antiplatelet or anticoagulant therapy
Tefferi et al. (2021)	Comparing bleeding incidences in extreme thrombocytosis (ExT, >1000 × 10⁹/L) and non‐ExT (<1000 × 10⁹/L) in low‐risk ET	Major bleeding: defined as bleeding events that require red cell transfusions	Incidence of major bleeding in ExT: 15% Incidence of major bleeding in non‐ExT: 10%	Sample size: 433 ET patients (183 with ExT, 250 without ExT)
Venkat et al. (2024)	Comparing bleeding incidences in extreme thrombocytosis (ExT, > 1000 × 10⁹/L) and non‐ExT (<1000 × 10⁹/L)	Major bleeding: ISTH definition (fatal, critical site, haemoglobin drop ≥2 g/dL, or transfusion of ≥2 units) CRNMB: bleeding requiring medical attention but not meeting major bleeding criteria	Cumulative incidence of all bleeding in ExT vs. non‐ExT patients: 21% vs. 13%. Cumulative incidence of all major bleeds in ExT vs. non‐ExT patients: 16% vs. 15%	Sample size: 451 ET patients (128 with ExT, 323 without ExT) Median follow‐up: ExT: 12 years Non‐ExT: 6 years
Wille et al. (2022)	Comparing bleeding‐free survival across myeloproliferative neoplasm subtypes	Major bleeding: defined as bleeding greater than II° (e.g., transfusion‐dependent anaemia, central nervous system involvement, retroperitoneal haemorrhage or other life‐threatening bleeding)	Major bleeding incidence: 6% Minor bleeding incidence: 12%	Sample size: 266 ET patients

### 1.2 What Are the Challenges to Managing Bleeding Complications in ET Patients?

The management of bleeding in JAK2‐variant ET is challenging for several reasons. First, there are inadequate risk stratification tools that predict which patients are at risk of bleeding. Commonly used risk stratification tools such as the IPSET‐t and its revised version, while useful for thrombosis risk prediction, have not been demonstrated to predict haemorrhage‐free survival in ET [10]. Platelet counts alone are inadequate predictors of bleeding as well [11, 18, 19]. Second, the acquired bleeding diathesis is frequently under‐recognised unless specifically tested for [16]. A patient may have subclinical abnormalities in von Willebrand factor (VWF) and platelet function that go unnoticed until an invasive procedure or trauma leads to an unexpected haemorrhage. Third, therapies to prevent thrombotic complications, such as low‐dose aspirin used routinely in many ET patients to reduce microvascular symptoms and clot risk, can tip the balance toward bleeding if an acquired VWF or platelet dysfunction is present. Lastly, the optimal strategy to correct the haemostatic defect is not well defined by current evidence. Therefore, clinicians must individualise management, balancing thrombosis prevention with measures to prevent bleeding.

## 2 Mechanisms of Bleeding in Essential Thrombocythemia

### 2.1 Acquired Von Willebrand Disease in Essential Thrombocythemia

**FIGURE 1:**
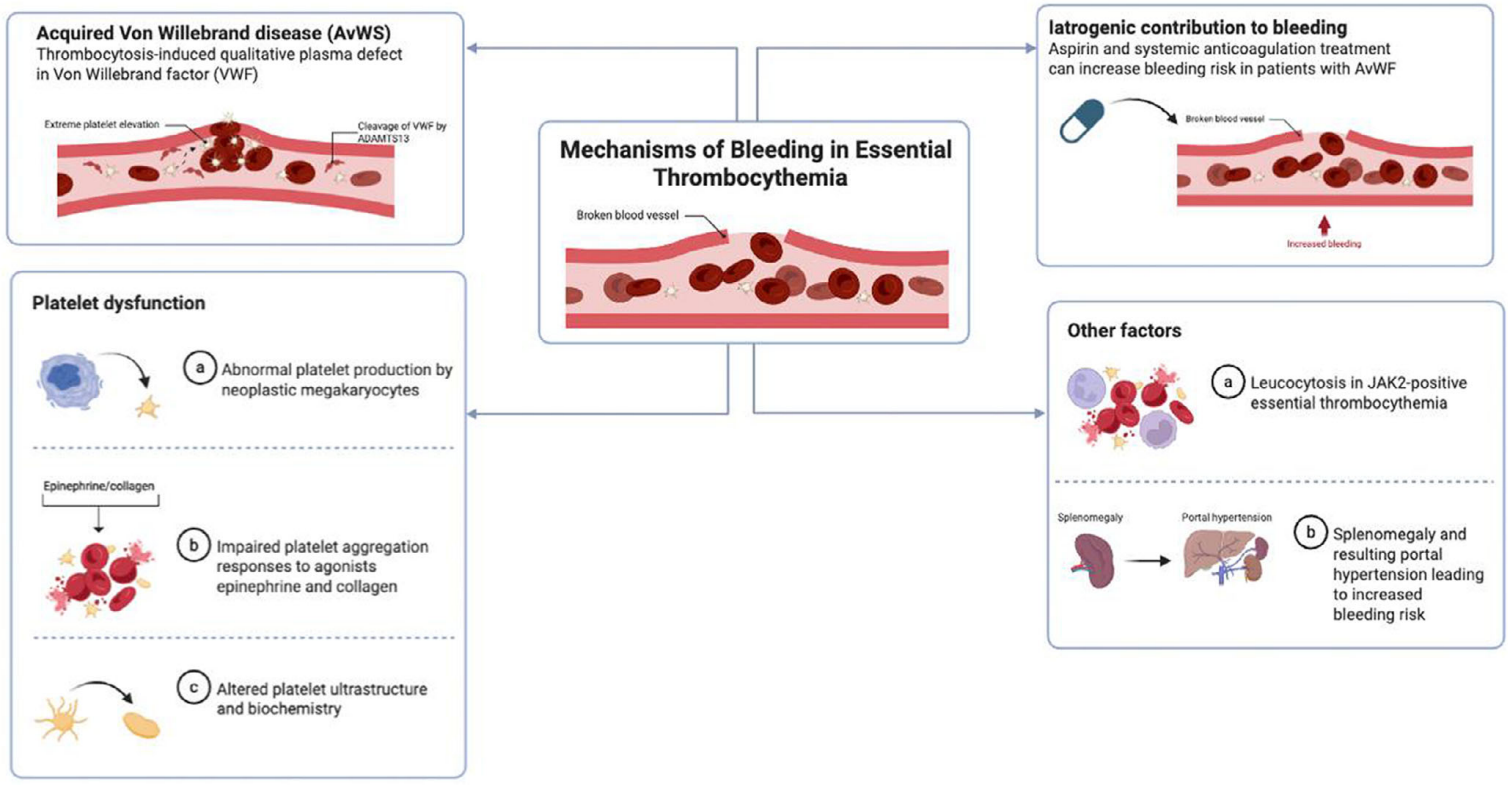
Mechanisms of bleeding in essential thrombocythemia (ET). Bleeding in ET arises from multiple interrelated mechanisms. Extreme thrombocytosis leads to a qualitative defect in von Willebrand factor (VWF), resulting in acquired von Willebrand syndrome (AvWS), often exacerbated by cleavage of VWF multimers by ADAMTS13. Platelet dysfunction stems from abnormal platelet production by neoplastic megakaryocytes, impaired aggregation responses, and altered platelet ultrastructure and biochemistry. Iatrogenic factors, such as aspirin and anticoagulation therapy, can unmask or worsen bleeding in patients with underlying AvWS. Additional contributors include leukocytosis, particularly in JAK2‐variant ET. (Figure created with BioRender)

The mechanism driving bleeding in ET is heterogeneous and not fully understood (Figure 1). The most well‐known cause is acquired von Willebrand syndrome (AvWS), precipitated by extreme elevation of platelets [15, 16, 20]. The pathophysiology involves both the adsorption of VWF onto platelets and the proteolysis of VWF multimers. In ET, when platelet counts are markedly elevated, the platelets can adsorb large VWF multimers out of circulation and promote their degradation [21]. Several studies have explored platelet turnover in patients with ET, most often using mean platelet volume (MPV) as a marker [22, 23, 24]. However, their association with bleeding remains inconclusive [25]. The high platelet mass provides an expanded surface for VWF to stick to and, as platelets bind VWF, the high‐molecular‐weight multimers of VWF are removed from the plasma and sometimes cleaved by the VWF‐cleaving protease ADAMTS13 [26]. The result is an acquired type 2A‐like VWD, characterised by decreased VWF activity despite normal or only mildly reduced VWF antigen levels [27]. Indeed, laboratory findings in ET‐associated AvWS mirror those of inherited type 2A VWD with low VWF ristocetin cofactor activity (VWF:RCo), a VWF:RCo to antigen ratio <0.7, and loss of the largest multimers on gel electrophoresis [16, 28]. In JAK2‐variant ET, this effect may be exacerbated by the presence of relatively higher haematocrit or other blood rheology changes promoting shear stress and VWF cleavage [21]. Importantly, these abnormalities and bleeding symptoms are noted to correct when the platelet count is reduced to normal, demonstrating that the VWF defect is secondary to thrombocytosis [29].

### 2.2 Platelet Dysfunction in Essential Thrombocythemia

Beyond AvWS, intrinsic platelet function abnormalities in ET also contribute to bleeding. The platelets produced in ET are often qualitatively abnormal due to the neoplastic megakaryocytes that generate them. Historical studies have documented a variety of platelet dysfunction in ET. For instance, bleeding time is prolonged in roughly 10–20% of patients, even in the absence of antiplatelet agents [30]. In vitro, platelet aggregation tests have shown inconsistent abnormalities such as impaired aggregation responses to agonists like epinephrine or collagen, and signs of platelet activation and degranulation in circulation [31, 32]. Platelet ultrastructure and biochemistry can be altered in ET as well. Some patients demonstrate a form of acquired storage pool deficiency, which in turn might not form an efficient haemostatic plug [33, 34, 35].

### 2.3 Association of JAK2 Variant and Bleeding

Patients harbouring a JAK2 variant are at increased risk of bleeding for several reasons. It is thought that JAK2 V617F not only alters platelet function but also remodels megakaryocyte biology. In murine models, the mutation enhances megakaryocyte migratory capacity, proplatelet extension, and proplatelet branching. This leads to an increased output of structurally and functionally abnormal platelets [36]. The model demonstrated that JAK2 V617F megakaryocytes yield platelets with altered receptor densities and secretory granule content, which directly contributes to the bleeding phenotype despite thrombocytosis. Complementary studies in JAK2 V617F knock‐in mouse models corroborate these findings, showing reduced thrombus formation and smaller thrombus size despite prothrombotic tendencies, underscoring the complexity of platelet dysfunction in this setting [37].

Beyond quantitative receptor changes, JAK2 V617F platelets exhibit qualitative deficiencies in receptor activation. Studies of ET patients have shown that, compared with CALR‐variants or triple‐negative cases, JAK2 V617F platelets have the lowest αIIbβ3 activation and P‐selectin expression in response to agonists, though whether this translates to an increased thrombohaemorrhagic outcome is less certain [38]. Further studies suggest that JAK2 V617F may modulate the phosphorylation status of signalling intermediates downstream of GPIb‐IX‐V engagement, weakening the initial adhesive steps of platelet plug formation [39]. Physiological levels of constitutive JAK2 signalling also skew the balance of pro‐ and anti‐aggregatory pathways, diminishing platelet responsiveness to submaximal stimuli [40]. Lastly, the variant may predispose a patient with ET to AvWS. It is thought that endothelial cells with the JAK2 V617F variant directly impair VWF synthesis and/or release [41]. In that study, JAK2 V617F‐expressing mice developed an acquired von Willebrand‐like disorder, characterised by a pronounced loss of high‐molecular‐weight VWF multimers and diminished ristocetin‐mediated platelet agglutination.

## 3 Predictors of the Risk of Bleeding in Essential Thrombocythemia

### 3.1 The Platelet Count‐Bleeding Correlation

In essence, thrombocytosis can induce a qualitative plasma defect in VWF. Historically, thrombocytosis >1000 × 10^9^/L was used as a surrogate for predicting the presence of biochemical type 2 AvWS, (indicated by vWF activity:antigen ratio being <60‐70% of normal). However, recent studies have shown that a substantial proportion of patients with platelets below this threshold also have significant reductions in VWF activity. One study characterising factors associated with the development of AvWS in ET determined that those harbouring the JAK2 V617F variant were more likely to have lower vWF activity levels for a given platelet count than those with the CALR variant. The median platelet count for developing AvWS in the JAK2‐variant cohort was 773 × 10^9^/L. In comparison, the median count for developing AvWS in the CALR variant cohort was 920 × 10^9^/L [16].

However, platelet count per se does not necessarily correlate directly with bleeding risk or adverse bleeding outcomes. While an analysis of the PT1 trial cohort suggested that platelet count > 450 × 10^9^/L (as opposed to within normal limits) was associated with an increased risk of major haemorrhage [42], several other studies noted no significant difference in major bleeding rates in ET patients with platelet counts <1000 × 10^9^/L versus >1000 × 10^9^/L [11, 18, 19]. While a higher platelet count was associated with the risk of developing AvWS, most patients present with platelet counts <1000 × 10^9^/L [11, 16, 28]. Clinicians should therefore be cautious when solely relying on platelet count to determine the risk of bleeding.

### 3.2 Use of Antiplatelet and Anticoagulants

By necessity, many patients with ET receive antiplatelet therapy. Aspirin, for example, is used to relieve microvascular symptoms and reduce arterial thrombosis risk in ET [43, 44]. In a patient who already may have an underlying platelet dysfunction or AvWS, the addition of aspirin can unmask or worsen bleeding tendencies [45]. The ARES trial found that twice‐daily low‐dose aspirin was superior to once‐daily low‐dose aspirin in addressing rapid platelet turnover and persistent microvascular symptoms [46]. Yet this regimen lacks prospective trial evidence comparing its risks and benefits for thrombohaemorrhagic complications against standard once‐daily therapy.

There is uncertainty regarding the role of antiplatelet therapy. While one study found that anticoagulation, rather than antiplatelet agents, was a risk factor, several others have identified aspirin as an independent predictor of bleeding in patients with a prior haemorrhage [10, 13, 47]. Aspirin dosing should therefore be individualised to the patient's risk [48].

Likewise, systemic anticoagulation given for treatment or prevention of thrombosis may increase bleeding risk. A retrospective series of patients with MPNs, including ET, who received direct oral anticoagulants (DOACs) report substantial bleeding incidences [49]. In the cohort of 133 MPN patients treated with DOACs (75 for VTE and 46 for atrial fibrillation), the 1‐year cumulative incidence of bleeding was 12.3% (95% CI: 6.4–18.2). Another real‐world study found that, over a median follow‐up of 1.7 years, major haemorrhagic complications were observed in 5.9% of those on DOACs [50].

By contrast, long‐standing use of vitamin K antagonists (VKAs) in MPN‐associated VTE has been linked with lower but still appreciable bleeding rates. In the European LeukemiaNet cohort, major bleeding on VKA occurred at 2.4 events per 100 patient years, with a cumulative probability of major bleeding at one year of 2.8% [51]. Notably, in a matched analysis of 71 MPN patients (45 on VKA, 26 on DOAC), there were no significant differences in either overall bleeding or major bleeding between the two anticoagulants [52].

### 3.3 Other Clinical Risk Factors

To our knowledge, no haemorrhage‐specific risk score has been developed or validated exclusively for ET patients. As mentioned, neither the IPSET‐T nor its revision predicts bleeding‐free survival [10]. Hence, clinical characteristics are crucial when identifying a patient with ET at risk of bleeding. Factors that have been implicated in bleeding risk include age >60 years, a prior bleeding history, splenomegaly, leucocytosis and diabetes mellitus [8, 11, 13, 53].

## 4 Screening for Acquired von Willebrand Disease in Essential Thrombocythemia

There is some debate as to whether all newly diagnosed patients with ET should be tested for acquired von Willebrand disease and platelet function abnormalities. On one hand AvWS in ET is not uncommon, being reported in up to 55% of patients [16, 28, 54]. Early identification of AvWS might therefore be beneficial in guiding management. The question is “Should a VWF screen be performed on a non‐urgent basis in all patients confirmed to have ET, even in the absence of bleeding symptoms?”. The rationale for this approach would be that many of these patients may be commenced on antiplatelet therapy which can exacerbate bleeding from AvWS. In addition, patients with ET, who are usually older individuals may require interventional procedures or surgical operations, which can lead to haemorrhagic complications if AvWS may have been missed.

On the other hand, routine testing of every ET patient for AvWS has cost implications and may not change management in asymptomatic individuals. Additionally, routine testing of all patients will detect mild VWF abnormalities that may never translate into clinical problems. For instance, one study of 64 patients with ET reported an incidence of AvWS at 41%. However, clinically significant bleeding was rare with only one patient in the study having a bleed. Hence, routine, universal screening may provide little benefit. Rather, targeted testing in high‐risk individuals may be a more judicious use of resources [55].

In practice, a middle‐ground recommendation might be screening for AvWS in ET in situations with a higher pretest probability or when results would immediately influence management. These situations include (1) marked thrombocytosis (>1000 × 10^9^/L), (2) the presence of bleeding symptoms even if the platelet counts are <1000 × 10^9^/L, or (3) the need for high‐risk interventions (e.g., surgery or other procedures where bleeding could be clinically problematic) [55].

Some clinicians may also factor in the genetic profile given that the JAK2‐variant is associated with a higher likelihood of AvWS [16].

## 5 Diagnosis of Acquired von Willebrand Disease in Essential Thrombocythemia

**FIGURE 2:**
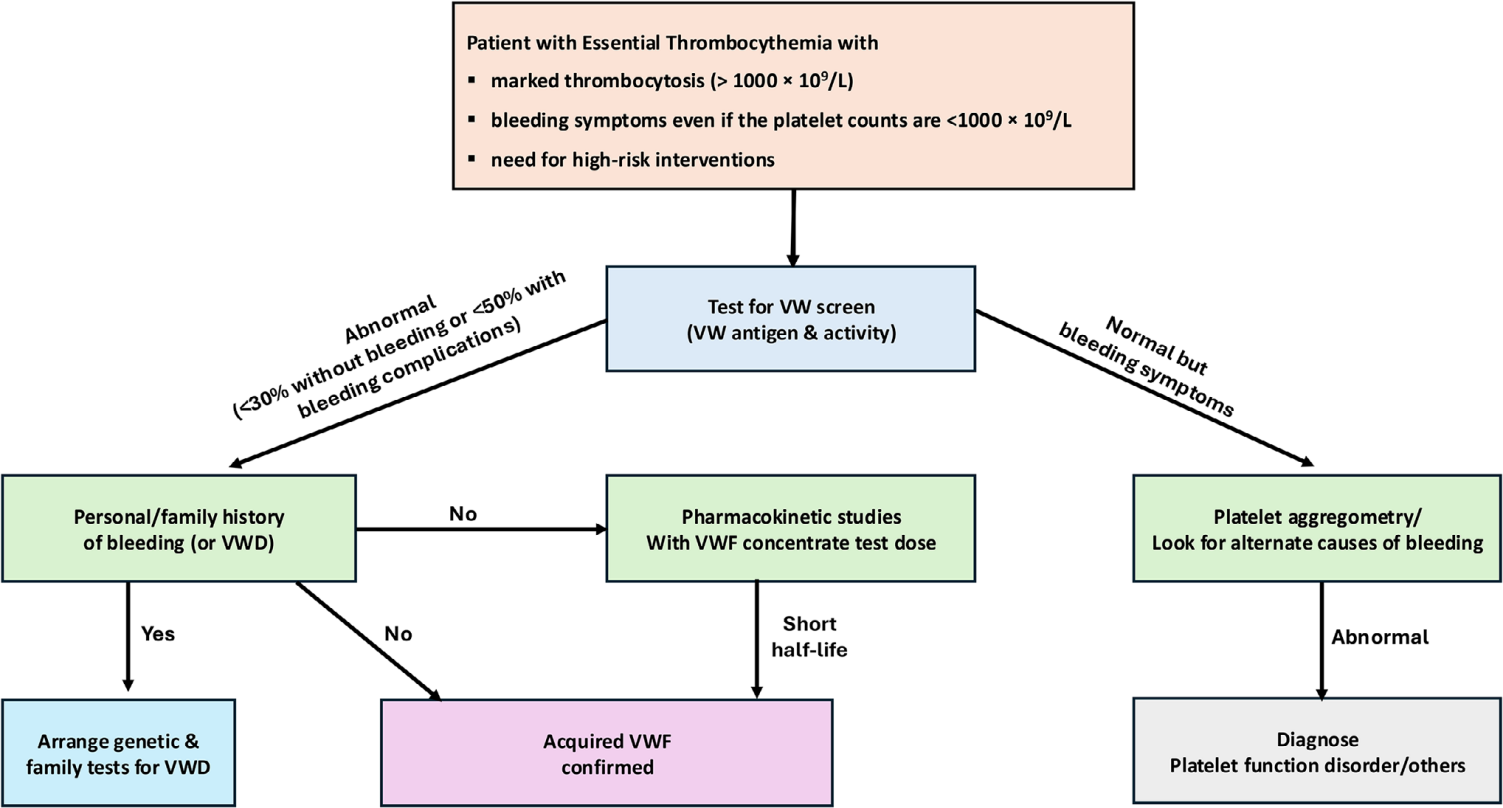
Diagnostic approach to bleeding in essential thrombocythemia with suspected acquired von Willebrand syndrome. Initial screening involves von Willebrand factor (VWF) antigen and activity levels. If acquired von Willebrand syndrome (AvWS) is confirmed, family history and genetic testing for inherited von Willebrand disease (VWD) should be considered. In patients with persistent bleeding but normal VWF studies, platelet function testing such as aggregometry may reveal alternative or coexistent causes. A VWF concentrate test dose with pharmacokinetic studies can help assess VWF clearance, guiding perioperative planning or replacement strategies.

The laboratory work‐up for AvWS mirrors that of inherited von Willebrand disease but with some nuances (Figure 2). Key tests include (1) VWF antigen (VWF:Ag), which measures the level of the protein, (2), VWF activity, traditionally measured by the ristocetin cofactor assay (VWF:RCo), and more recently, using VWF binding to platelet glycoprotein assays,  and (3) factor VIII coagulant activity (FVIII:C) [56, 57]. In patients with ET and AvWS, VWF antigen is often normal or mildly decreased, but VWF activity is reduced out of proportion to the antigen, leading to a low activity‐to‐antigen ratio (typically <0.5–0.7) [58, 59]. A key diagnostic test described in the published literature is VWF multimer analysis on gel electrophoresis, which in acquired VWD due to ET usually shows loss of the largest multimers [60, 61, 62, 63], leading to the type 2A pattern previously mentioned. In practice, not all laboratories can perform multimer analysis readily, so the combination of a low VWF activity and low VWF activity:antigen ratio in a patient with ET with thrombocytosis may point to the diagnosis of AvWS. It is useful in this context to ask about personal and family history of bleeding, since the presence of family members with bleeding symptoms or bleeding complications before the ET diagnosis may suggest congenital VWD.

## 6 Diagnosis of Platelet Dysfunction in Essential Thrombocythemia

Platelet function disorders in ET are not as common as AvWS. If a patient has bleeding symptoms but VWF testing is normal, one may consider formal platelet aggregometry to look for an intrinsic platelet defect bearing in mind that the responses are highly variable and may vary in the same patient over time [64, 65]. Traditional light transmission aggregometry may show reduced aggregation to ADP, collagen and epinephrine (39%, 37% and 57% of cases respectively) [66]. It has been suggested that decreased or absent aggregation in response to epinephrine by comparison with other agonists is classical for MPD and may be attributed to a decrease in the number of platelets α2‐adrenergic receptors [67].

Still, routine platelet function testing is not recommended for all patients with ET as it has not been shown to correlate well with bleeding risk and does not change management in most cases. If a patient continues to have bleeding issues despite normalised VWF levels, specialised testing for platelet granular content or release reactions might be undertaken to identify rare coexistent platelet storage pool deficiencies or secretion defects (PSPDs). Platelet transfusions in PSPDs are infrequently required and are generally ineffective for certain granule defects. In general, platelet transfusion should be reserved for life‐threatening bleeding and bleeding that not is managed by adjuvant therapy such as antifibrinolytics and desmopressin [68]. Prophylactic transfusion is generally discouraged in δ‐SPD patients outside of life‐threatening haemorrhage [69].

## 7 Management of Acute Bleeding

Management of acute bleeding in patients with ET‐associated AvWS must focus on rapid restoration of haemostatic function. Plasma‐derived VWF/factor VIII concentrates are the first‐line treatment for severe haemorrhage. In the ISTH registry, administration of VWF/FVIII concentrates at doses of 30–100 U/kg of VWF:RCo achieved clinical haemostasis in approximately 40% of AvWS cases, although the shortened half‐life of infused VWF in myeloproliferative contexts necessitates repeated dosing every 6–8 h until bleeding control is achieved [70]. It is essential to monitor VWF activity and factor VIII levels to guide interval dosing and ensure sustained haemostatic levels.

Desmopressin (DDAVP) is ineffective in severe types 1 and 2A AvWS, whilst also being contraindicated in type 2B [71, 72]. One international registry study noted that it has poor efficacy in patients with MPN, with only around 20% having bleed control [59]. Case series and clinical experience report effective haemostasis in 60–80% of minor bleeding episodes with antifibrinolytics agents when used alone or in combination with DDAVP or VWF concentrates [73, 74]. In cases of platelet dysfunction in MPNs, platelet transfusions may be considered in cases of life‐threatening bleeding, or prior to procedures, due to the lack of functional platelets in circulation [68].

### 7.1 Management Conundrums—Should Aspirin be Given in the Setting of AvWS in ET?

Low‐dose aspirin (typically 75 mg/day) is recommended for all patients with JAK2 mutated ET to reduce arterial thrombosis risk, with a twice‐daily schedule generally reserved for those with problematic microvascular symptoms [75, 76, 77]. Aspirin reduces the risk of arterial thrombosis by inhibiting platelet aggregation through irreversible cyclooxygenase‐1 (COX‐1) inhibition. Conversely, AvWS increases bleeding risk and aspirin may further exacerbate this by inhibiting platelet function. The use of aspirin in patients with thrombocytosis >1000 × 10^9^/L therefore requires monitoring for bleeding events and clinically relevant AvWS [78]. In general, there is a lack of prospective or clinical trial data to guide aspirin use in those with AvWS. It is advisable to screen for ristocetin cofactor activity when platelet counts exceed 1000 × 10^9^/L. If activity is below 20–30%, withholding aspirin should be strongly considered to avoid unmasking severe AvWS and amplifying bleeding risk [75]. Consideration could also be given to delaying the initiation of aspirin until cytoreductive therapy lowers platelet counts to <1000 × 10⁹/L in high‐risk cases [55, 75].

### 7.2 Management Conundrums—Should Cytoreduction be Considered in the Setting of AvWS in ET?

Although cytoreductive therapy is generally guided by IPSET and several guidelines’ criteria [75, 76, 79], it may also be warranted in patients who experience major or clinically significant nonmajor bleeding, or in asymptomatic individuals with extreme thrombocytosis (>1000 × 10^9^/L) and confirmed biochemical AvWS [80, 81]. That said, one study found no clear benefit of cytoreduction for bleeding risk based solely on that platelet threshold [11]. As mentioned, the mechanisms behind bleeding in those with ET are heterogeneous and not fully understood. Without any definite guidelines, the decision to undertake cytoreduction should be individualised, taking into account the high‐risk factors discussed earlier.

If the decision to cytoreduce is undertaken, hydroxycarbamide is often the first‐line agent for most patients with ET and is effective in reducing platelet counts [82], thereby correcting the acquired VWD. Another cytoreductive option is interferon‐alpha. There is a paucity of data on interferon's impact on AvWS in ET, but by lowering platelet count it may be considered to have the same beneficial effect. Two trials have compared hydroxycarbamide and pegylated interferon as frontline options for reducing platelet count in ET. The MPN‐RC 112 trial, which enrolled newly diagnosed patients with high‐risk polycythaemia vera and ET, demonstrated that both treatments achieved comparable complete response rates at 12 months based on ELN criteria. However, while hydroxycarbamide was associated with a higher rate of histopathologic response, pegylated interferon was noted for its sustained reduction in the JAK2‐variant allele burden over time [83].

Anagrelide is an imidazo‐quinazolin compound known to inhibit platelet aggregation [84]. In ET, anagrelide reduces platelet count by targeting both megakaryocyte hyperproliferation and differentiation through incompletely understood mechanisms [85]. The PT‐1 randomised trial, which compared hydroxyurea plus aspirin versus anagrelide plus aspirin in patients with high‐risk ET, found that the anagrelide arm had more serious haemorrhage events [86]. However, in the phase III ANAHYDRET trial using the updated 2008 WHO criteria for ET, it was shown to be noninferior in terms of bleeding when compared with hydroxyurea [87]. In practice, deciding on the first‐line cytoreductive agent should involve a discussion with patients regarding the expected haematologic outcomes, the distinct toxicity profiles of these agents, and the limited data on differences in disease progression [80].

## 8 Periprocedural Management of Patients with ET and AvWS

Patients with ET are noted to have a predisposition to an increased risk of bleeding during surgery [73]. Appropriate perioperative management is therefore crucial. For a patient with ET known to have AvWS, the ideal strategy for an elective major procedure is to correct the VWF defect prior to surgery [88, 89]. There are two general ways to do this: lower the platelet count thus allowing endogenous VWF levels to recover and/or provide exogenous VWF.

Lowering the platelet count is typically accomplished via cytoreductive agents given prior to the procedure. If time permits (the procedure is not urgent), one can initiate or intensify cytoreductive therapy to bring the platelet count down toward normal, thereby correcting the AvWS. There are reports of using urgent high‐dose hydroxycarbamide in the acute setting to reduce platelet counts rapidly. For instance, one case report explored a high‐dose hydroxyurea regimen (3 g/day) when a patient with ET developed severe bleeding at the time of a bone marrow biopsy. Within 24 h the platelet count decreased by half from 1500 × 10^9^/L to 750 × 10^9^/L and the bleeding ceased [90]. Interestingly, platelet function parameters were also normalised. Alternatively, plateletpheresis can be used for immediate reduction of platelet count if the situation is urgent, although this remains a rarely used option [91]. However, its effects are temporary, and as such generally reserved for life‐threatening situations or when cytoreductive drugs are contraindicated. The American Society for Apheresis, for example, recommends plateletpheresis as a second‐line therapy in symptomatic thrombocytosis [92].

The second approach, providing an exogenous von Willebrand factor, is often necessary if surgery is imminent or if the VWF activity remains low despite platelet reduction. The mainstay is VWF concentrates with dosing typically guided by the degree of VWF deficiency and the invasiveness of the procedure and can be similar to that in the case of congenital VWD [88]. For major surgeries, repeated doses or continuous infusion to maintain haemostatic levels for several days postoperatively may be required [89]. One of the key issues in this respect where the patients may be different to individuals with congenital VWD is the need for more frequent VWF concentrates, whereby the patients may require up to three times a day dosing regimen (versus twice daily in the case of congenital VWD). Regular monitoring of the VWF levels is also crucial and hence major procedures may require to be undertaken in hospitals with specialist laboratories.

### 8.1 Management Conundrums—Should Thromboprophylaxis be Given in the Setting of AvWS in ET?

Prophylactic anticoagulation postsurgery in patients with ET requires special consideration. Patients with a JAK2 variant are already at increased risk of thrombosis and surgery may exacerbate this risk [93, 94]. In general, VTE prophylaxis should be considered in this patient group but a balance between the risks of thrombosis and bleeding must be carefully assessed [95, 96]. If the patient presents without clinical characteristics associated with high bleeding risk, as discussed earlier, a low threshold is maintained for giving VTE prophylaxis [97]. For those with high bleeding risk or those who may develop bleeding while receiving pharmacological thromboprophylaxis, mechanical prophylaxis should be considered. One study determined that low‐dose aspirin was associated with a reduced incidence of venous thromboembolism with no effect on the risk of bleeding in patients with JAK2‐variants [98]. The same was not noted in the CALR‐variant. Larger prospective studies are needed before recommendations can be made for thromboprophylaxis in this group. Until then we suggest an individualised approach with informed patient discussion.

## 9 Conclusion

Bleeding in ET highlights the paradox where thrombocytosis leads to impaired haemostasis through AvWS and platelet dysfunction. This is particularly common in those with a JAK2 variant. Clinicians must monitor for bleeding and test for VWF abnormalities. Extreme thrombocytosis sometimes requires cytoreductive therapy even in patients traditionally deemed low risk for thrombosis. Careful planning of periprocedural haemostasis is also crucial in this group. Further research is needed to refine risk stratification. In the meantime, a personalised approach to monitoring and intervention remains key to bleeding control.

## Author Contributions

Gerard Gurumurthy: Literature search and writing — original draft. Samantha Gurumurthy: Literature search and writing — original draft. Tim C. P. Somervaille: Writing — review & editing. Anna Falanga: Writing — review & editing. Jecko Thachil: Writing — review & editing and conceptualisation.

## Conflicts of Interest

The authors declare no conflicts of interest.

## Data Availability

Not applicable.
